# Rates of clinical remission and inadequate response to advanced therapies among patients with ulcerative colitis in Germany

**DOI:** 10.1007/s00384-023-04397-7

**Published:** 2023-05-08

**Authors:** Bernd Bokemeyer, Nils Picker, Daniel Kromer, Ludger Rosin, Haridarshan Patel

**Affiliations:** 1 Interdisciplinary Crohn Colitis Centre Minden, North Rhine-Westphalia, Märchenweg 17, 32429 Minden, Germany; 2 Real World and Advanced Analytics, Ingress-Health HWM GmbH, Wismar, Mecklenburg-Vorpommern Germany; 3 Medical Affairs, Galapagos Biopharma Deutschland GmbH, Munich, Bavaria, Germany; 4 Evidence Generation and Epidemiology, Medical Affairs, Galapagos NV, Mechelen, Antwerp, Belgium

**Keywords:** Ulcerative colitis, Real-world treatment, Inadequate response, Advanced therapy

## Abstract

**Purpose:**

Many patients treated for ulcerative colitis (UC) do not achieve clinical remission. This real-world study assessed clinical remission and inadequate response rates among patients with UC in Germany treated with advanced therapies.

**Methods:**

This retrospective chart review included patients with UC newly initiating advanced (index) therapy (anti-TNFα agents, vedolizumab, tofacitinib) from January 2017–September 2019 (index date). Included patients had data for ≥ 12 months before (baseline period) and after the index date (follow-up period). Remission was defined as a partial Mayo score ≤ 1. Indicators of inadequate response were: index therapy discontinuation; therapy adjustments (index therapy dose escalation; augmentation with non-advanced therapies; corticosteroid [CS] use during maintenance therapy); CS dependency (use for ≥ 12 weeks); and UC-related hospitalisation, surgery or emergency department visit. Time to first remission and inadequate response were analyzed using Kaplan–Meier analyses.

**Results:**

Among 149 patients with UC (median age: 40 years), 96 (64.4%) were biologic-naïve and 42 (28.2%) received CS at the index date. Within 12 months, 52 patients (47.2%) were in remission; of these, 13 patients (25.0%) received ≥ 1 therapy adjustment. At 12 months, 55 patients (37.6%) had ≥ 1 indicator of an inadequate response. Median time to remission was longer among biologic-experienced vs biologic-naïve patients (24 vs 7 months; *p* = 0.012).

**Conclusion:**

Over half of the patients were not in clinical remission after 12 months and more than one-third experienced inadequate response. One-quarter of patients in remission required therapy adjustments. Patients with UC require therapies that are more effective than those currently available to achieve better treatment outcomes.

**Supplementary Information:**

The online version contains supplementary information available at 10.1007/s00384-023-04397-7.

## Introduction

Ulcerative colitis (UC) is a chronic idiopathic inflammatory bowel disease experienced by approximately 150 000 patients in Germany [1] and is associated with a high patient and economic burden [2−5]. The therapeutic goals for patients with moderately to severely active UC are to achieve and maintain long-term corticosteroid-free clinical and endoscopic remission, and to improve their health-related quality of life. Conventional therapies, such as corticosteroids (CS), 5-aminosalicylic acid (5-ASA), and immunosuppressive agents (e.g. azathioprine), are used to treat inflammation and control UC-related symptoms. Therapeutic options for patients who do not respond to conventional therapies include biologic therapies such as anti-tumor necrosis factor alpha (TNFα), anti-integrin and anti-interleukin agents, as well as orally-administered small molecule therapies such as Janus kinase (JAK) inhibitors and ozanimod, a sphingosine-1-phosphate receptor modulator [1, 6, 7].

Although the treatment options for patients with moderate to severe UC have expanded in the past few years, several recent real-world studies have reported a high proportion of patients not being in clinical remission and/or experiencing suboptimal response to treatment with advanced therapies. For example, in one real-world study involving approximately 200 patients with UC, two-thirds of patients receiving anti-TNFα, and half of patients receiving vedolizumab did not achieve clinical remission after 6 months of treatment [8]. Furthermore, corticosteroid-free clinical remission for at least 3 months was achieved by fewer than a quarter of patients with UC in a smaller real-world study involving approximately 100 patients [9]. Dose escalation of biologic treatments, a common real-world treatment strategy upon secondary loss of response to biologic treatments in patients with UC [10], occurred in more than half of biologic-treated patients in one real-world study that involved 300 patients [11], which also reported concomitant use of CS in three-quarters of patients [11]. Additionally, CS dependency was reported in approximately 15% of patients in a large, multicenter study involving more than 2000 patients [12]. Finally, augmentation of advanced therapies with conventional therapies, switching to other advanced treatments and therapy discontinuation in cases of insufficient response have been frequently observed in multiple real-world studies [11, 13−15].

In this study, we aimed to assess clinical remission and inadequate response rates in patients with UC who received advanced treatment in real-world practice in Germany. We also aimed to explore factors associated with clinical remission and inadequate response.

## Materials and methods

### Study design and data collection

This multicenter, retrospective medical chart review describes the proportion of patients with UC in clinical remission and with indicators of inadequate response in routine German clinical practice. More than 400 specialist outpatient gastroenterology practices nationwide with experience in the advanced treatment of patients with IBD were invited to participate in this study at random. In total, 18 study centres from 10 geographic regions of Germany expressed willingness to participate and were included. The median number of patients with UC treated across these sites before data extraction for the study began was 250 per year (range: 40–1200). The study centres performed the data extraction from a subset of preselected patients in order to achieve balanced group sizes with respect to the advanced therapies initiated.

Data were extracted using a web-based electronic case report form to capture data in a standardized format. Before the study started, all site investigators received detailed information on the background, study objectives, and regulatory obligations. Regular contact with study sites was maintained throughout the data collection process to resolve data entry discrepancies with input logic and query missing data fields.

Eligible patients were required to be 18 years or older with a confirmed diagnosis of UC, based on the German and European Crohn’s and Colitis UC diagnostic guidelines [1, 16]. Patients were included in the analysis if they initiated UC-related treatment with an advanced therapy between January 2017 and September 2019. The agents considered advanced therapies for UC management were anti-TNFα agents (infliximab, adalimumab, and golimumab), tofacitinib, and vedolizumab. No influence was exerted on the advanced treatment that a patient initiated; the treatment decision was taken completely independently between patient and gastroenterologist at the participating study sites in accordance with UC treatment guidelines.

All eligible patients had to be included in an initial screening list. Patients were randomly selected from this list to achieve an even distribution of patients with respect to the index therapies received (anti-TNFα, anti-integrin, or JAK inhibitor). No further predefined criteria were considered for the selection of patients from the initial screening list. Up to 15 eligible patients were selected from each participating study site.

Patients who received biologics/JAK inhibitors for non-UC conditions (e.g. Crohn’s disease, indeterminate colitis, psoriasis arthritis, or rheumatoid arthritis) during the whole study period were excluded from the study. In addition, patients who underwent a total colectomy before the start of an advanced treatment were excluded.

Data were extracted between January and May 2021 for patients with a minimum of 12 months of available data before (baseline period) and after the index date (follow-up period). The index date was defined as the date on which patients were prescribed a new advanced therapy and was considered the start of follow-up. The study outcomes were investigated for the duration of the individual patient follow-up periods, ending at death, discontinuation of index therapy, or end of follow-up, whichever came first.

### Study outcomes

Baseline patient demographics recorded included age at the start of index therapy and sex. Baseline clinical characteristics at index date included: disease duration; prior use of both non-advanced and advanced therapies during the 12-month baseline period; frequency of stools per day, rectal bleeding, endoscopic findings, Physician’s Global Assessment (PGA) of disease severity, C-reactive protein, and fecal calprotectin levels in the 4 weeks prior to the index date (if available) were also recorded.

The primary outcomes assessed were clinical remission and indicators of inadequate response. Clinical remission was defined as the achievement of a partial Mayo score of not more than 1. The Mayo sub-scores for stool frequency, rectal bleeding, and disease activity based on the PGA were used to calculate the partial Mayo scores for each patient with available data [17]. The assessments for disease activity were performed at 3, 6, 12 and 24 months following the initiation of advanced therapy. Indicators of inadequate response were defined as at least one of the following events: index therapy discontinuation; therapy adjustments (dose escalation of index therapy; augmentation with non-advanced therapies; any CS use during the maintenance phase [defined as the period from 12 weeks after index date until end of follow-up period]); CS dependency (defined as use for > 12 weeks); and UC-related hospitalization, surgery, or emergency department visit. The earliest identified occurrence of one of these events determined the time of first observed inadequate response. Augmentation was defined as a newly prescribed non-advanced therapy such as 5-ASA, systemic and locally acting CS, azathioprine, mercaptopurine, methotrexate, or a therapy with ciclosporin or tacrolimus that was not used at the index date.

The secondary outcomes were an evaluation of the factors associated with time to the first achievement of clinical remission and indicators of inadequate response. Furthermore, rates of discontinuation as well as dose escalation of index therapy were analyzed. The reasons for therapy discontinuation, according to the prespecified categories, included: ‘worsening or unsatisfactory control of UC-related symptoms’; ‘acute drug-related reaction or adverse events’; ‘switch to another advanced therapy’; ‘patient’s decision to terminate the advanced treatment’; ‘development of neutralizing anti-drug antibodies by the patient’; or ‘surgical intervention’. The mean weekly dose at the start of the maintenance phase of each index therapy was assessed. Additionally, the proportion of patients with concurrent use of CS while initiating index therapy and during the maintenance phase was described, as well as the number of patients with CS dependency. Finally, the frequency of adverse events while receiving the index therapy was also assessed.

For the analysis of baseline characteristics, clinical remission, inadequate response, index therapy discontinuation, dose escalation and CS dependency, patients were also stratified into all or some of the following subgroups according to: prior use of biological therapy (biologic-experienced vs biologic-naïve), use of CS at the index date, and clinical remission status in the first 12 months after the index date.

### Statistical analysis

Patient characteristics were analyzed using descriptive statistics for the overall population and the predefined subgroups. Absolute and relative frequency tabulations described categorical variables. Mean, standard deviation, median, and interquartile range (IQR) were reported for continuous variables. Furthermore, time-to-event analyses were performed using the Kaplan–Meier method accounting for individual follow-up periods, censoring patients at the end of the observed period. The median time to event and 95% confidence interval (CI) were reported for the overall population and the predefined subgroups.

Comparisons between subgroups were made by using the Chi-square test for categorical variables, unpaired *t*-test and Wilcoxon rank-sum test for continuous variables, and the log-rank test for time-to-event estimates.

Multivariate Cox proportional hazards models were used to estimate the factors associated with time to remission and time to experience an indicator of inadequate response to advanced therapy. Both models included the following covariates: age at index; sex; index therapy; partial Mayo sub-score; prior use of biological therapy before the index date; active use of CS at index date; and concurrent use of other non-advanced therapies at the start of index therapy. The impact on the time to event was estimated for each covariate, and the respective hazard ratios (HRs) and related 95% CIs were reported.

The data collection and management were carried out using MySQL version 8.0 and statistical analyses were done in Stata version 17.0 (StataCorp LLC, College Station, TX, USA).

## Results

### Patient baseline characteristics

In total, 149 patients with UC were included in this study; approximately half of patients were female (Table 1). The median age at study inclusion was 40 years (IQR: 30–54) and the median disease duration was 6.3 years (IQR: 2.2–12.8). The median post-index follow-up period was 25.9 months (IQR: 20.4–38.5). According to the available baseline period, 96 patients (64.4%) were biologic-naïve, whereas 51 patients (34.2%) were biologic-experienced, and of these patients, 47 (92.2%) were previously treated with anti-TNFα agents. Among included patients, 75 (50.3%) received an anti-TNFα agent as index therapy, 48 (32.2%) vedolizumab, and 26 (17.4%) tofacitinib. The partial Mayo score was significantly higher among biologic-experienced patients than among patients without prior exposure to biologic treatments (mean: 6.2 vs 5.4; *p* < 0.05). Concomitant use of CS at initiation of index therapy was identified in fewer than one-third of the patients (*n* = 42, 28.2%).

**Table 1:** Baseline demographics and characteristics of the overall sample of patients initiating an advanced therapy for the management of UC as well as predefined subsamples

**Demographic/characteristic**	**Overall**	**Prior use of biologics**^**a**^			**Use of CS at start** **of index therapy**		
		**Biologic-naïve**	**Biologic-experienced**	***p* value**	**Yes**	**No**	***p* value**
Number of patients	149 (100)	96 (64.4)	51 (34.2)		42 (28.2)	107 (71.8)	
Age at index, years							
Mean (SD)	42.2 (15.5)	42.8 (15.7)	41.2 (15.0)	0.539	46.5 (16.0)	40.6 (15.0)	0.042
Median (IQR)	40 (30–54)	41 (29–54)	39 (30–54)	0.647	49 (31–59)	38 (28–53)	0.035
Sex							
Male	74 (49.7)	47 (49.0)	26 (51.0)	0.815	23 (54.8)	51 (47.7)	0.436
Female	75 (50.3)	49 (51.0)	25 (49.0)		19 (45.2)	56 (52.3)	
Partial Mayo score	*N* = 137	*N* = 89	*N* = 46		*N* = 40	*N* = 97	
Mean (SD)	5.6 (1.7)	5.4 (1.6)	6.2 (1.7)	0.018	6 (1.5)	5.5 (1.7)	0.069
Median (IQR)	6 (5–7)	6 (4–7)	7 (5–7)	0.011	6 (5–7)	6 (4–7)	0.097
Stool frequency	*N* = 145	*N* = 93	*N* = 50		*N* = 41	*N* = 104	
Normal number of stools	5 (3.5%)	3 (3.2%)	1 (2.0%)	0.035	0 (0%)	5 (4.8%)	0.344
1–2 stools more than normal	21 (14.5%)	18 (19.4%)	3 (6.0%)		4 (9.8%)	17 (16.4%)	
3–4 stools more than normal	52 (35.9%)	36 (38.7%)	15 (30.0%)		16 (39.0%)	36 (34.6%)	
5 or more stools more than normal	67 (46.2%)	36 (38.7%)	31 (62.0%)		21 (51.2%)	46 (44.2%)	
Rectal bleeding	*N* = 143	*N* = 92	*N* = 49		*N* = 40	*N* = 103	
No blood seen	30 (21.0%)	22 (23.9%)	7 (14.3%)	0.164	4 (10.0%)	26 (25.2%)	0.098
Streaks of blood with stools in most cases	49 (34.3%)	34 (37.0%)	14 (28.6%)		18 (45.0%)	31 (30.1%)	
Obvious blood with stools most of the time	61 (42.7%)	35 (38.0%)	26 (53.1%)		18 (45.0%)	43 (41.8%)	
Blood alone passed	3 (2.1%)	1 (1.1%)	2 (4.1%)		0 (0%)	3 (2.9%)	
PGA	*N* = 142	*N* = 92	*N* = 48		*N* = 42	*N* = 100	
Normal	2 (1.4%)	1 (1.1%)	1 (2.1%)	0.492	0 (0%)	2 (2.0%)	0.152
Mild disease	17 (12.0%)	11 (12.0%)	5 (10.4%)		4 (9.5%)	13 (13.0%)	
Moderate disease	92 (64.8%)	63 (68.5%)	28 (58.3%)		24 (57.1%)	68 (68.0%)	
Severe disease	31 (21.8%)	17 (18.5%)	14 (29.2%)		14 (33.3%)	17 (17.0%)	
Endoscopic findings	*N* = 102	*N* = 72	*N* = 29		*N* = 28	*N* = 74	
Normal mucosa or inactive disease	5 (4.9%)	2 (2.8%)	3 (10.3%)	0.220	0 (0%)	5 (6.8%)	0.055
Mild activity	16 (15.7%)	11 (15.3%)	4 (13.8%)		1 (3.6%)	15 (20.3%)	
Moderate activity	47 (46.1%)	37 (51.4%)	10 (34.5%)		14 (50.0%)	33 (44.6%)	
Severe activity	34 (33.3%)	22 (30.6%)	12 (41.4%)		13 (46.4%)	21 (28.4%)	
CRP value,^b^ mg/L	*N* = 120	*N* = 81	*N* = 38		*N* = 39	*N* = 81	
Mean (SD)	15.5 (18.7)	16.4 (19.8)	13.7 (16.3)	0.431	17.8 (22.1)	14.3 (16.8)	0.383
Median (IQR)	9 (3–19)	9 (3–22)	9 (3–15)	0.861	10 (2–24)	8 (3–16)	0.771
FC level,^b^ μg/g	*N* = 77	*N* = 45	*N* = 31		*N* = 29	*N* = 48	
Mean (SD)	922 (925.8)	1023.6 (937.0)	802.8 (910.0)	0.308	1174.8 (1075.0)	769.3 (796.5)	0.085
Median (IQR)	600 (322–1101)	725 (447–1204)	600 (275–800)	0.093	800 (323–1781)	600 (311–802)	0.092
Index therapy							
Anti-TNFα agent	75 (50.4%)	64 (66.7%)	10 (19.6%)	-	19 (45.2%)	56 (52.3%)	-
Vedolizumab	48 (32.2%)	27 (28.1%)	20 (39.2%)	-	16 (38.1%)	32 (29.9%)	-
Tofacitinib	26 (17.4%)	5 (5.2%)	21 (41.2%)	-	7 (16.7%)	19 (17.8%)	-
Non-advanced UC therapies at baseline^c^							
Any CS^d^	95 (64.6%)	61 (63.5%)	34 (66.7%)	0.706	42 (100%)	53 (49.5%)	< 0.001
5-aminosalicylic acid	108 (72.5%)	74 (77.1%)	33 (64.7%)	0.215	32 (76.2%)	76 (71.0%)	0.526
Azathioprine	49 (32.9%)	34 (35.4%)	14 (27.5%)	0.542	11 (26.2%)	38 (35.5%)	0.276
Other non-advanced therapies^e^	6 (4.0%)	4 (4.2%)	2 (3.9%)	0.956	0 (0%)	6 (5.6%)	0.117
Duration of UC, years	*N* = 136	*N* = 84	*N* = 51		*N* = 39	*N* = 97	
Mean (SD)	8.3 (7.9)	7.8 (7.8)	9.3 (8.1)	0.305	8.8 (9.8)	8.2 (7.0)	0.732
Median (IQR)	6.3 (2.2–12.8)	4.8 (2.0–12.6)	7.1 (2.6–13.4)	0.136	5.3 (1.9–13.4)	6.7 (2.3–12.2)	0.552
Follow-up after index, months							
Mean (SD)	28.8 (10.3)	29.0 (10.1)	28.2 (10.4)	0.638	29.6 (10.4)	28.5 (10.2)	0.559
Median (IQR)	25.9 (20.4–38.5)	26.9 (21.0–38.4)	24 (19.8–39.0)	0.518	27.2 (20.2–39.1)	24.7 (20.4–38.5)	0.541

*Bio* biologic, *CRP* C-reactive protein, *CS* corticosteroid, *FC* fecal calprotectin, *IQR* interquartile range, *PGA* Physician’s Global Assessment, *SD* standard deviation, *UC* ulcerative colitis

^a^Data for the clinical parameters assessed as part of the baseline characteristics were not available for all patients; in these cases, the number of patients with available data is indicated by *N*. Unless otherwise specified, all characteristics are n (%), where the percentage is given out of the number patients for whom data were available. History of prior use of biologics was not available for two patients. These two patients also did not have any treatment history data available for use of non-advanced UC therapies

^b^CRP and FC levels were reported for values in the 4 weeks before the index date

^c^UC-related non-advanced therapies were reported for the 12-month baseline period

^d^Including systemic and locally acting CS

^e^Other non-advanced therapies included mercaptopurine, methotrexate and ciclosporin or tacrolimus

### Primary outcomes

#### Achievement of clinical remission

**Fig. 1:**
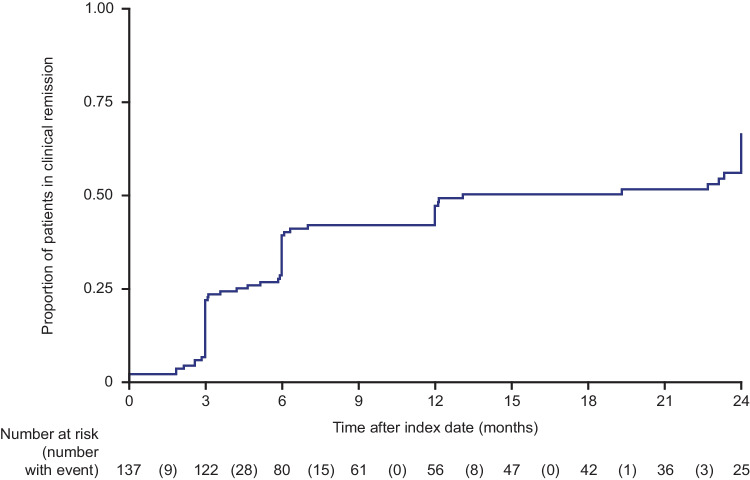
Time to first clinical remission among patients with UC treated with advanced therapy. Patients without a partial Mayo score at baseline (*n* = 12) were excluded *UC*, ulcerative colitis

Among patients with an available partial Mayo score of more than 1 at baseline (*n* = 134), 52 (47.2%) were in remission within 12 months after initiation of index therapy (median time to first remission: 13.1 months [95% CI: 7–24]) (Fig. 1). Among patients in remission within 12 months after advanced therapy initiation, 13 (25.0%) required at least one adjustment of the index therapy, including dose escalation of index therapy, augmentation with non-advanced therapies, CS-dependency, or use of CS during the maintenance phase.

**Fig. 2:**
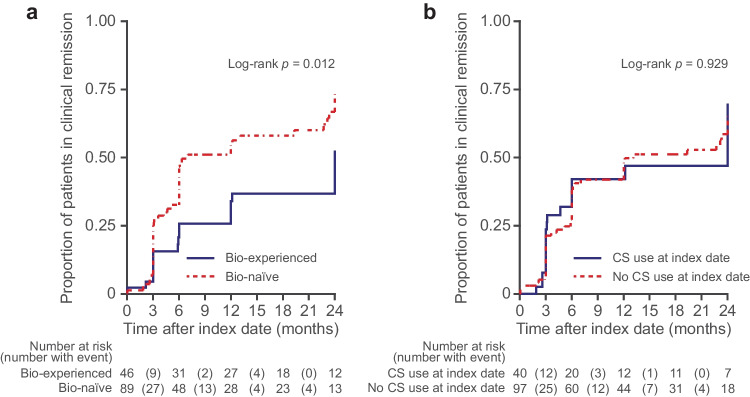
Time to first clinical remission among patients with UC treated with advanced therapy by (**a**) previous biologic use^a^ and (**b**) CS use at index date^b^ ^a^Patients without a partial Mayo score at baseline (*n* = 12) were excluded. Patients with incomplete medication history information were also excluded (*n* = 2) ^b^Patients without a partial Mayo score at baseline (*n* = 12) were excluded *Bio*, biologic; *CS*, corticosteroid; *UC*, ulcerative colitis

The median time to achieve clinical remission was significantly longer among biologic-experienced patients than biologic-naïve patients (24 months vs 7 months; log-rank *p* = 0.012) (Fig. 2a). No differences were found among patients with and without active use of CS at the index date (Fig. 2b).

#### Inadequate response to advanced therapies

**Fig. 3:**
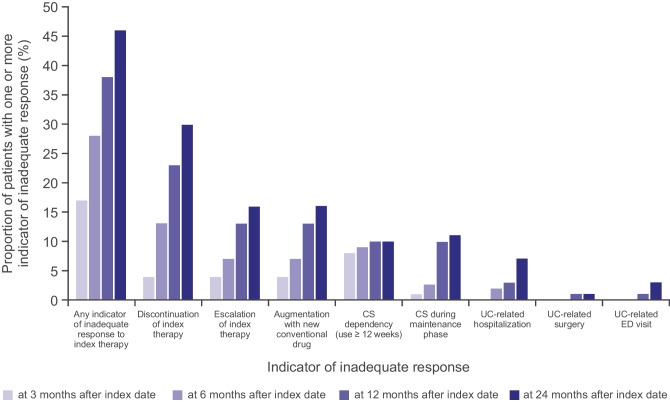
Proportion of patients with inadequate response to index therapy by indicator measured at 3, 6, 12 and 24 months *CS*, corticosteroid; *ED*, emergency department; *UC*, ulcerative colitis

**Fig. 4:**
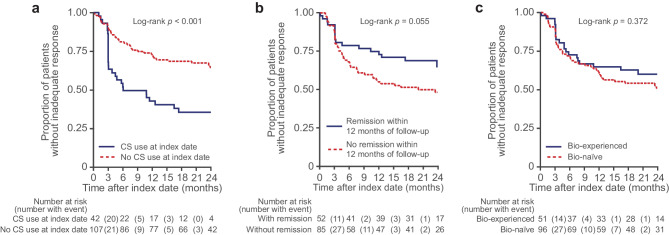
Time to any indicator of inadequate response among patients with UC treated with advanced therapy by (**a**) CS use at index date, (**b**) remission status within the first 12 months of follow-up^a^ and (**c**) previous biologic use ^a^Patients without a partial Mayo score at baseline (*n* = 12) were excluded *Bio*, biologic; *CS*, corticosteroid; *UC*, ulcerative colitis

Among all patients in the study, 55 (37.6%) experienced at least one indicator of inadequate response at 12 months after initiation of index therapy (Fig. 3). Indicators of inadequate response were detected earlier in patients with active use of CS than in those who were not using CS at index (at 12 months, 59.5% vs 29.0%; log-rank *p* < 0.001) (Fig. 4a). Patients who did not achieve remission within 12 months were also found to have earlier indicators of inadequate response versus those who did (at 12 months, 47.5% vs 24.6%; log-rank *p* = 0.055) (Fig. 4b). No differences were observed for rates of inadequate response between biologic-experienced and biologic-naïve patients (Fig. 4c).

### Secondary outcomes

#### Factors associated with time to remission and inadequate response

In an adjusted Cox regression model (Table 2), prior use of advanced therapies (HR: 0.49; 95% CI: 0.26–0.95; *p* = 0.034) was associated with a lower likelihood of achieving clinical remission, and use of non-advanced therapies at index was associated with a higher probability of achieving clinical remission (HR: 1.82; 95% CI: 1.09–3.02; *p* = 0.021).

**Table 2:** Multivariate Cox regression model for first achievement of clinical remission during index therapy

**Covariate**	**HR (95% CI)**	***p*** **value**
Age at index date ≥ 40 years (*n* = 68)	0.926 (0.57–1.52)	0.759
Female (*n* = 68)	0.861 (0.52–1.41)	0.554
Index therapy		
Anti-TNFα (*n* = 68)	Reference	
Vedolizumab (*n* = 41)	1.061 (0.61–1.85)	0.835
Tofacitinib (*n* = 24)	1.619 (0.70–3.74)	0.259
Partial Mayo score	0.849 (0.71–1.02)	0.082
Prior advanced therapy (*n* = 45)	0.491 (0.26–0.95)	0.034
Active use of CS at index date (*n* = 40)	0.615 (0.34–1.12)	0.109
Other active non-advanced therapies at index date^a^ (*n* = 75)	1.818 (1.09–3.02)	0.021

Multivariate Cox regression model estimating factors associated with the likelihood of observing first achievement of clinical remission among patients with UC treated with advanced therapies (*n* = 135)

*CI* confidence interval, *CS* corticosteroid, *HR* hazard ratio, *TNFα* tumor necrosis factor alpha, *UC* ulcerative colitis

^a^Non-advanced therapies included 5-aminosalicylic acid, azathioprine, mercaptopurine, methotrexate and tacrolimus or ciclosporin

According to the multivariate analysis, patients who were older than 40 years at index were at lower risk of experiencing an inadequate response than younger patients (HR: 0.55; 95% CI: 0.33–0.91; *p* = 0.021). Compared with the use of anti-TNFα agents as index therapy, vedolizumab was associated with a lower risk of inadequate response (HR: 0.46; 95% CI: 0.25–0.86; *p* = 0.014), whereas tofacitinib was not (HR: 0.53; 95% CI: 0.23–1.25; *p* = 0.148). Higher partial Mayo score at baseline (HR: 1.24; 95% CI: 1.04–1.47; *p* = 0.016) and active use of CS at index (HR: 2.58; 95% CI: 1.53–4.36; *p* < 0.001) were associated with a higher risk of inadequate response (Table 3).

**Table 3:** Multivariate Cox regression model for first inadequate response to advanced therapies

**Covariate**	**HR (95% CI)**	***p*** **value**
Age at index date ≥ 40 years (*n* = 69)	0.545 (0.33–0.91)	0.021
Female (*n* = 69)	0.776 (0.47–1.29)	0.327
Index therapy		
Anti-TNFα (*n* = 68)	Reference	
Vedolizumab (*n* = 43)	0.462 (0.25–0.86)	0.014
Tofacitinib (*n* = 24)	0.532 (0.23–1.25)	0.148
Partial Mayo score	1.236 (1.04–1.47)	0.016
Prior advanced therapy (*n* = 46)	1.168 (0.63–2.17)	0.624
Active use of CS at index date (*n* = 40)	2.582 (1.53–4.36)	< 0.001
Other active non-advanced therapies at index date^a^ (*n* = 76)	1.430 (0.84–2.43)	0.186

Multivariate Cox regression model estimating factors associated with the probability of observing indicators of inadequate response among patients with UC treated with advanced therapies (*n* = 135)

*CI* confidence interval, *CS* corticosteroid, *HR* hazard ratio, *TNFα* tumor necrosis factor alpha, *UC* ulcerative colitis

^a^Non-advanced therapies included 5-aminosalicylic acid, azathioprine, mercaptopurine, methotrexate and tacrolimus or ciclosporin

#### Discontinuation of advanced therapies

Overall, 34 patients (23%) had discontinued the index therapy at 12 months after treatment initiation. Among the 47 patients who discontinued their index therapy within the entire follow-up period, 80.9% switched to another advanced treatment. Higher rates of discontinuation of index therapy were observed among patients with active use of CS than patients without active use of CS at index (at 12 months, 34% vs 18%; log-rank *p* = 0.044). No significant difference was identified when comparing patients based on their experience with prior advanced therapies (at 12 months, 26% in biologic-naïve vs 18% in biologic-experienced patients; log-rank *p* = 0.246).

The primary reasons for discontinuing the index therapy are shown in Supplemental Table 1. The most frequent reason was the worsening or unsatisfactory control of UC signs and symptoms (*n* = 27; 57.4%), followed by a drug-related acute reaction or an adverse event from the advanced therapy (*n* = 5; 10.6%). In total, 26 adverse events were reported among patients (Supplemental Table 2), the most frequent of which was anemia (*n* = 10; 38.5%), followed by drug intolerance (*n* = 3; 11.5%).

#### Dose escalation of advanced therapies

At the start of the maintenance phase, the mean initial dosages were infliximab 48 mg/week (*n* = 31), adalimumab 20 mg/week (*n* = 31), golimumab 22 mg/week (*n* = 8), vedolizumab 40 mg/week (*n* = 45), and tofacitinib 112 mg/week (*n* = 25).

At 12 months, 16 patients (12.7%) received an escalation of the index therapy. During the follow-up period, dose escalation mainly occurred due to the worsening or unsatisfactory control of UC symptoms (17 of 21 patients; 85%). Other reasons for treatment escalation were low drug concentration (*n* = 1), skin disease (pyoderma; *n* = 1), and preventive escalation due to anticipated severe course of disease (*n* = 1). No differences in rates of dose escalation were observed within the predefined subgroups.

#### Concurrent use of CS and assessment of CS dependency

At index, systemic CS were prescribed for 36 patients (24.2%), locally acting CS for eight patients (5.4%), and two patients (1.3%) received both systemic and locally acting CS. At 12 months after the index date, 13 patients (10.4%) had received CS during the maintenance phase of the advanced therapy, and CS dependency was observed in 14 patients (10.1%). Rates of CS dependency were significantly higher among patients with active use of CS at index compared with those without (at 12 months, 25.6% vs 4.1%; log-rank *p* < 0.001).

## Discussion

This retrospective, multicenter, medical chart review collected clinical data across all regions of Germany from biologic-experienced and biologic-naïve patients with UC who newly initiated advanced therapies. As a result, this study presents new evidence that is reflective of routine German clinical practice.

In our study, nearly half of the observed patients did not achieve remission in the 12 months after advanced therapy initiation. This in line with what has been reported in a similar retrospective chart review of patients with UC in Germany [8], which defined clinical remission as a total Mayo score ≤ 2 compared with a partial Mayo score ≤ 1 in the present study. In our study, we further found that patients with prior exposure to biologic treatments were less likely to achieve remission than biologic-naïve patients, whereas active use of non-advanced therapies at index date was associated with a greater likelihood of being in remission. The use of CS at the index date, however, was not associated with a greater likelihood of being in remission. As well as this, we report that among patients in remission 12 months after index therapy initiation, one-quarter received a therapy adjustment.

Inadequate response after 12 months of index therapy initiation was found to occur in one-third of patients in this study. This high rate of inadequate response is broadly in line with that observed in a similarly designed multinational cohort study and a German retrospective claims data study, which reported that an even higher 50% and 75% of patients with UC experienced inadequate response within the first 12 months of treatment, respectively [14, 15]. Furthermore, our study found that patients with active use of CS at initiation of index therapy experienced an indicator of inadequate response earlier than patients without active use of CS. Moreover, we showed that rates of inadequate response to index therapy were higher among patients who did not achieve remission within 12 months than among patients who did.

Discontinuation and dose escalation of index therapy were the most frequently reported indicators of inadequate response in our study, in line with previous findings [14, 15].

We report that discontinuation occurred in one-quarter of patients at 12 months, and the proportion of patients who discontinued was even higher among those with active use of CS at index date. Additionally, we found that dose escalation occurred in one out of 10 patients at 12 months, although other retrospective studies using claims data at the patient level have shown dose escalation to be more common, with approximately half of patients with UC having received a dose increase of more than 50% after 12 months [4, 11]. Discontinuation and escalation of index therapy in our study were primarily due to for the worsening and unsatisfactory control of UC-related symptoms, and drug-related adverse events, similar to what has been reported in other recent studies [9, 14].

We observed CS dependency in one out of 10 patients at 12 months after therapy initiation and this proportion was significantly higher among patients with active use of CS at index than among those without CS use at index. In addition, our study showed that at 12 months, 10% of patients used CS during maintenance. In contrast, the use of CS was found to be much more widespread in a recent German claims data study that analyzed CS prescriptions not only from gastroenterologists, but also from general practitioners and other specialists found [11]. In that study, approximately three-quarters of patients required CS during the follow-up period after initiation of a treatment with a biologic, one-quarter of patients used CS for more than 14 weeks within a 24-month follow-up period, and approximately one out of ten patients newly initiated CS in the follow-up period [11]. Another claims data study, found the prolonged use of steroids within the first year of treatment was 36% [15]. It is possible that our study may have underestimated the use of CS in routine clinical practice, given that the collected data were limited to specialized gastroenterology centers with experience in advanced treatment of patients with IBD. Therefore, this study did not take into account possible additional prescriptions of CS by general practitioners, unlike the claims data analyses mentioned above. The differences in study design may explain the discrepancy between the reported use of CS among the studies.

One of the main strengths of this study is the multicenter design based on real-world data, which reflects the therapeutic management of UC in routine clinical practice in Germany. Indeed, 18 study centers from a total of 10 geographic regions participated and these were reasonably well-distributed between urban and rural areas (defined as having a population of ≥/< 50,000 inhabitants; rural: 7/18 sites, 47/149 [31.5%] of patients). Moreover, compared with administrative database analyses, the assessment of disease activity through chart review is more reliable and accurate. Furthermore, data extraction was performed for all patients at the study sites who met the inclusion and exclusion criteria; thus, potential selection biases associated with consent-based research studies were avoided. Finally, patients were selected from the initial screening list to achieve an even distribution with respect to the advanced index therapy received. In this way, potential bias for selecting convenient patients to study was avoided.

Nonetheless, the findings of this retrospective chart review should be interpreted with caution. Although this study included patients treated in outpatient gastroenterology practices throughout Germany, only one of the 18 participating sites was located in Southern Germany. Likewise, from the participating study sites, only a relatively small number of patients were eligible for medical record review based on the prespecified selection criteria. In addition, although sites were randomly selected, only those that expressed interest participated in the study, which might have led to selection bias. Furthermore, the remission and inadequate response data might have been incomplete owing to some patient data recorded at outpatient practices being restricted. However, unlike previous analyses of administrative databases [15], this medical chart review provides detailed insight into clinically relevant parameters, such as assessment of disease activity, over the course of treatment. Finally, the irregularity of information entry in medical records could increase the risk of observing a lack of data to analyze health resource use outcomes and adverse events.

Overall, we have shown that over half of patients with UC treated with advanced therapies in routine German clinical practice did not achieve clinical remission after 12 months, and half of patients required more than 1.5 years to achieve clinical remission. Among patients who were in remission 12 months after advanced therapy initiation, one-quarter required additional therapy adjustments. Finally, one-third of patients with UC experienced an indicator of inadequate response to advanced therapy at 12 months after having started treatment. Higher rates of inadequate response were observed in patients without remission and with concurrent use of CS at baseline.

Based on these findings, we conclude that there is a need for advanced treatments that enable a greater proportion of patients with UC to achieve better outcomes more quickly after treatment initiation and to maintain these outcomes in the long term.

### Supplementary Information

Below is the link to the electronic supplementary material.

Supplementary file1 (DOCX 24 KB)

## Data Availability

The anonymized data that support the findings of this study are available on request from the corresponding author.
