# Supplementary material for: Rates of clinical remission and inadequate response to advanced therapies among patients with ulcerative colitis in Germany
Source: Int J Colorectal Dis. 2023 May 8;38(1):116. doi: 10.1007/s00384-023-04397-7 (PMC10164668; doi:10.1007/s00384-023-04397-7)
Supplement: Supplementary file 1 — Supplementary file1 (DOCX 24 KB) [file 384_2023_4397_MOESM1_ESM.docx]

**Supplementary information**

Rates of clinical remission and inadequate response to advanced therapies among patients with ulcerative colitis in Germany

Bernd Bokemeyer^1^, Nils Picker^2^, Daniel Kromer^2^, Ludger Rosin^3^, Haridarshan Patel^4^

^1^ Interdisciplinary Crohn Colitis Centre Minden, Minden, North Rhine-Westphalia, Germany

^2^ Real World and Advanced Analytics, Ingress-Health HWM GmbH, Wismar, Mecklenburg-Vorpommern, Germany

^3^ Medical Affairs, Galapagos Biopharma Deutschland GmbH, Munich, Bavaria, Germany

^4^ Evidence Generation and Epidemiology, Medical Affairs, Galapagos NV, Mechelen, Antwerp, Belgium

**Corresponding author**

Prof. Dr. Bernd Bokemeyer

Interdisciplinary Crohn Colitis Centre Minden, Märchenweg 17, 32429 Minden, Germany

Tel: 0571/580998

E-mail: bernd.bokemeyer@t-online.de

**Plain Language Summary**

There are approximately 150,000 people in Germany who have ulcerative colitis (UC). UC is an inflammatory bowel disease that affects the gut lining. Although many UC treatments are available, many patients do not achieve clinical remission or do not have a good response to treatment. Clinical remission in UC means an improvement in the key signs and symptoms, such as reductions in rectal bleeding and stool frequency. Patients with UC who do not have a good response often change their treatment. Patients may stop their treatment, have their dose increased, or add corticosteroids to their treatment. However, long-term corticosteroid use is linked with side effects.

In this study, we used real-world medical history data taken from 149 patients from 18 bowel disease clinics in Germany. We calculated the percentage of patients with clinical remission and the percentage of patients who did not have a good response. The study included patients who had started an ‘advanced’ UC therapy between January 2017–September 2019. Advanced therapies target specific molecules involved in UC disease.

We found that:

- just over 50% of patients did not achieve remission within 12 months after starting advanced treatment.
- 25% of patients who were in remission within 12 months made treatment changes.
- more than one-third of patients did not have a good response 12 months after starting advanced treatment.

Our results suggest that patients with UC need treatments that are more effective than those currently available. This would help more patients achieve long-term improvements in UC signs and symptoms.

**Supplemental Table 1** Primary reason for discontinuation of index therapy

| **Primary reason** | ***N* = 47** |
| --- | --- |
| UC signs and/or symptoms worsened or were not controlled satisfactorily | 27 (57.4) |
| Patient experienced a drug-related acute reaction/AE from the advanced therapy | 5 (10.6) |
| Switch to another advanced UC-related therapy | 5 (10.6) |
| Patient decision to terminate the advanced therapy | 4 (8.5) |
| Patient developed neutralizing antibodies | 4 (8.5) |
| Patient required a surgical intervention to manage UC | 1 (2.1) |
| Tumor disease/urothelial carcinoma | 1 (2.1) |

Data are all n (%)

AE, adverse event; UC, ulcerative colitis

**Supplemental Table 2** Types of adverse events among patients with UC treated with advanced therapies

| **Adverse event** | ***N* = 26** |
| --- | --- |
| Anemia | 10 (38.5) |
| Drug intolerance | 3 (11.5) |
| Stroke or myocardial infarction | 1 (3.8) |
| Herpes zoster | 1 (3.8) |
| Upper respiratory tract infection | 1 (3.8) |
| Psoriasis | 1 (3.8) |
| Pneumonia | 1 (3.8) |
| Bacterial infection | 1 (3.8) |
| Hematological malignancy | 1 (3.8) |
| Bone fracture | 1 (3.8) |
| Hypertension | 1 (3.8) |
| Cholangitis | 1 (3.8) |
| Fatigue | 1 (3.8) |
| Circulatory problems, dizziness | 1 (3.8) |
| Urothelial carcinoma | 1 (3.8) |

Data are all n (%)

UC, ulcerative colitis
